# A Novel Dermocosmetic Routine Containing Vitamin B3 and 2‐Mercaptonicotinyl Glycine Significantly Improves Melasma After 3 Months of Daily Use

**DOI:** 10.1111/jocd.70476

**Published:** 2025-10-02

**Authors:** Juliane Rocio, Natalia Kovylkina, Tania Cestari, Mariana Fajgenbaum Feiges, Erika Fernandes, Claire Deloche‐Bensmaine, Elisa Caberlotto, Thierry Passeron

**Affiliations:** ^1^ Institute of Dermatology & Aesthetics of Rio de Janeiro Rio de Janeiro Brazil; ^2^ Vichy Laboratoires International Vichy France; ^3^ Department of Dermatology Hospital de Clinicas de Porto Alegre Porto Alegre Brazil; ^4^ L'Oréal LDB Rio de Janeiro Brazil; ^5^ Department of Dermatology, CHU Nice Université Côte d'Azur Nice France; ^6^ C3M, INSERM U1065 Université Côte d'Azur Nice France

**Keywords:** 2‐Mercaptonicotinyl glycine, Kligman's formula, Melasma, vitamin B3

## Abstract

**Introduction:**

Melasma is a chronic refractory pigmentation disorder. Kligman triple (KT) formula is the gold standard. A dermocosmetic (DC) serum containing vitamin B3 and 2‐mercaptonicotinyl glycine has been developed for melasma. This study assessed the benefits of DC serum compared to KT in melasma.

**Material and Methods:**

A randomized, single‐blind 6‐month study was conducted in women with melasma. Subjects were randomized to Group A (morning: serum; evening: cream, for 6 months) or to Group B (1st 3 months: morning: skin hydrating gel; evening: KT, followed by 3 months of DC routine regimen); all subjects applied a sunscreen with an SPF 50+ twice daily. Assessments included MASI, mMASI, IGA, skin hydration, radiance and fine wrinkles, tolerance, and quality of life (QoL) using MELASQOL.

**Results:**

Ninety‐one women aged 44 ± 6 years of Phototypes III (30%) and IV (36%) were recruited. Both regimens significantly (*p* < 0.05) improved MASI after 3 months. A clinically relevant difference in favor of KT was observed. Three months after the switch, both Group A (38.1%) and Group B (41.2%) provided a similar significant (*p* < 0.05) percentage reduction of MASI score, with no between‐group difference. The mMASI and IGA showed similar results. QoL and skin hydration significantly (*p* < 0.05) improved in both groups after 6 months. Skin radiance and fine wrinkles also improved. Global tolerance was better with the DC routine.

**Conclusions:**

Both DC routine and KT significantly improved melasma after 3 months of use. DC further improved melasma when replacing KT for 3 further months, with similar results when used alone for 6 months.

## 1 Introduction

Melasma is a frequently observed chronic skin hyperpigmentation requiring continued management and photoprotection throughout the year [1]. It presents with symmetrically disposed brown macules and patches, mainly in women between 20 and 50 years of age, individuals with Fitzpatrick's phototypes III–V, and subjects from multiple and mixed ethnicities [1, 2]. Even though not life‐threatening, melasma impacts the subjects' quality of life (QoL) [3].

Several factors participate in its etiopathogenesis, including genetic predisposition, hormonal factors, and frequent sunlight exposure [2, 4, 5]. Moreover, an increasing quantity of data suggests that melasma is related to early photoaging in predisposed individuals [1].

Skin aging is caused by intrinsic and extrinsic pathways. Genetic and hormonal factors are inherently associated with unavoidable skin aging, while environmental factors, such as ultraviolet radiation and visible light, are extrinsically associated with preventable skin aging [6, 7, 8, 9]. Recently, cellular senescence has been suggested to be a key player in skin‐aging‐related hyperpigmentation [10]. In addition, different skin cell types, such as keratinocytes, melanocytes, fibroblasts, and endothelial cells, play a role in skin aging. Their crosstalk during the aging process may play an important role in melanogenesis and the subsequent aging‐related pigmentation [11, 12].

Currently, several topical products are proposed to manage melasma [13, 14]. The most popular and efficient melasma treatment is certainly Kligman's formula (KT), which combines a corticosteroid (dexamethasone or fluocinolone), hydroquinone (HQ), and retinoic acid [13]. However, due to safety concerns (primarily exogenous ochronosis caused by HQ), KT is not indicated for the continued long‐term management of melasma [15, 16].

Recently, a dermocosmetic (DC) routine combining a serum containing vitamin B3 and 2‐mercaptonicotinyl glycine (Liftactiv Specialist B3 Serum, Laboratoires Vichy, France) was developed to treat melasma.

This study assessed the benefits of a DC routine in melasma, including DC serum and a cream (Liftactiv Night Cream, Laboratoires Vichy, France) compared to KT (Vitacid Plus Cream containing fluocinolone, tretinoin, and HQ, Theraskin Brazil).

## 2 Material and Methods

The randomized, single‐blind 6‐month study received approval from the Ethics Committee of the Pro‐Cardiac Hospital (6.721.734 issued on March 24, 2024). The study complied with the principles of the Declaration of Helsinki and Good Clinical Practices. All subjects provided written informed consent prior to inclusion.

The study was conducted in adult women with symmetrical epidermal melasma clinically diagnosed under Wood's light examination and lasting for more than a year. Assessments at all visits included scoring of the Melasma Area Severity Index (MASI), its modified version (mMASI), Investigator Global Assessment (IGA, 0 = clear to 5 = very severe), skin radiance and fine wrinkles (both 0 = none to 7–9 = very severe), local tolerance, and quality of life (QoL) using MELASQOL [17, 18, 19]. Cosmeticity was assessed by the subject on a 5‐point scale at each post‐baseline study visit. Skin hydration was assessed using a corneometer (Courage & Khazaka, Germany). Photos were taken for illustration purposes at all study visits.

After 4 weeks of wash‐out, subjects were randomized to Group A (DC routine: morning: serum; night: cream) or to Group B (first 3 months: morning: skin hydrating gel (Mineral 89, Laboratoires Vichy, France); evening: KT, followed by 3 months DC routine regimen); all subjects also applied a sunscreen with an SPF 50+ (UV Age Daily, Laboratoires Vichy, France) twice daily.

The primary endpoints of this study were the improvement of MASI and mMASI scores at Month 3 and Month 6.

Quantitative variables were summarized using means, medians, and standard deviation (SD). Qualitative variables were summarized as counts and percentages. For each parameter and treatment group, a graphical representation of the means±95% CI was produced to visually assess the evolution across time. The percentage change at timepoint (after baseline, Month 0) was calculated on the mean value observed for each parameter (where applicable) and treatment group. For MASI, mMASI score, and MelasQOL, Student's t‐test for paired samples and the Wilcoxon Signed Rank test were used. For the IGA for hyperpigmentation and clinical scores, the Wilcoxon Signed Rank test and Mann–Whitney *U*‐test were applied. The null hypothesis was rejected if a *p*‐value less than 0.05 (5% significance level) was produced by the statistical procedure. Microsoft Excel 2010 and IBM SPSS version 19.0 were used for the statistical analysis.

## 3 Results

In total, 91 adult women were recruited (Group A: 46; Group B: 45), with a mean age of 45 ± 5 years in Group A and 44 ± 6 years in Group B. They were mainly Phototypes III (29%) and IV (37%). Demographic and melasma baseline data are given in Table 1. Both groups were similar concerning the distribution of age, phototype, MASI, mMASI, and IGA scores.

**TABLE 1:** Demographic and baseline data.

		Group A (DC routine)	Group B (KT)
		*n* = 45	*n* = 46
Age	Mean ± SD	45 ± 5	44 ± 6
	Median	44	44
	Min; Max	37; 59	26; 62
	Total	46	45
Gender	Female	46 (100%)	45 (100%)
	Total	46 (100%)	45 (100%)
Phototype	I	0 (0%)	0 (0%)
	II	6 (13%)	6 (13%)
	III	14 (30%)	12 (27%)
	IV	17 (37%)	17 (38%)
	V	9 (20%)	10 (22%)
	VI	0 (0%)	0 (0%)
	Total	46 (100%)	45 (100%)
MASI	Mean ± SD	14.0 ± 4.7	14.3 ± 6.4
	Median	12.6	14.0
	Total	46 (100%)	45 (100%)
mMASI	Mean ± SD	6.4 ± 2.5	7.2 ± 3.6
	Median	6.0	7.3
	Total	46 (100%)	45 (100%)
IGA Hyperpigmentation	Mean ± SD	3.1 ± 0.8	3.6 ± 0.9
	Median	3.0	4.0
	Total	46 (100%)	45 (100%)
MELASQOL	Mean ± SD	44.4 ± 18.1	45.1 ± 16.9
	Median	45.5	47.0
	Total	46 (100%)	45 (100%)

**FIGURE 1:**
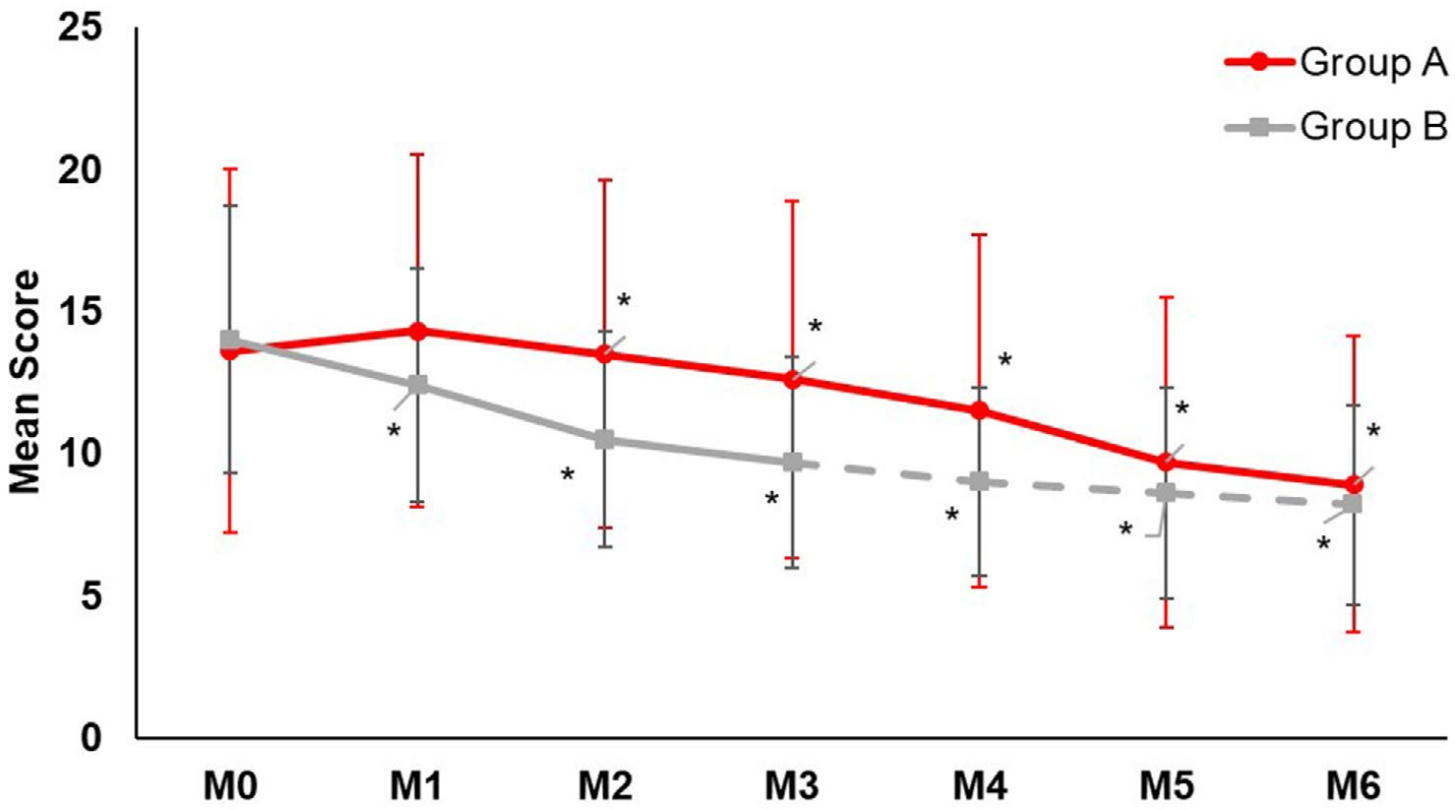
Mean MASI score over time. The decrease of the MASI score from baseline was statistically significant in both groups after 3 and 6 months. The between‐group difference was not statistically significant at any time point. *p ≤ 0.05.

**FIGURE 2:**
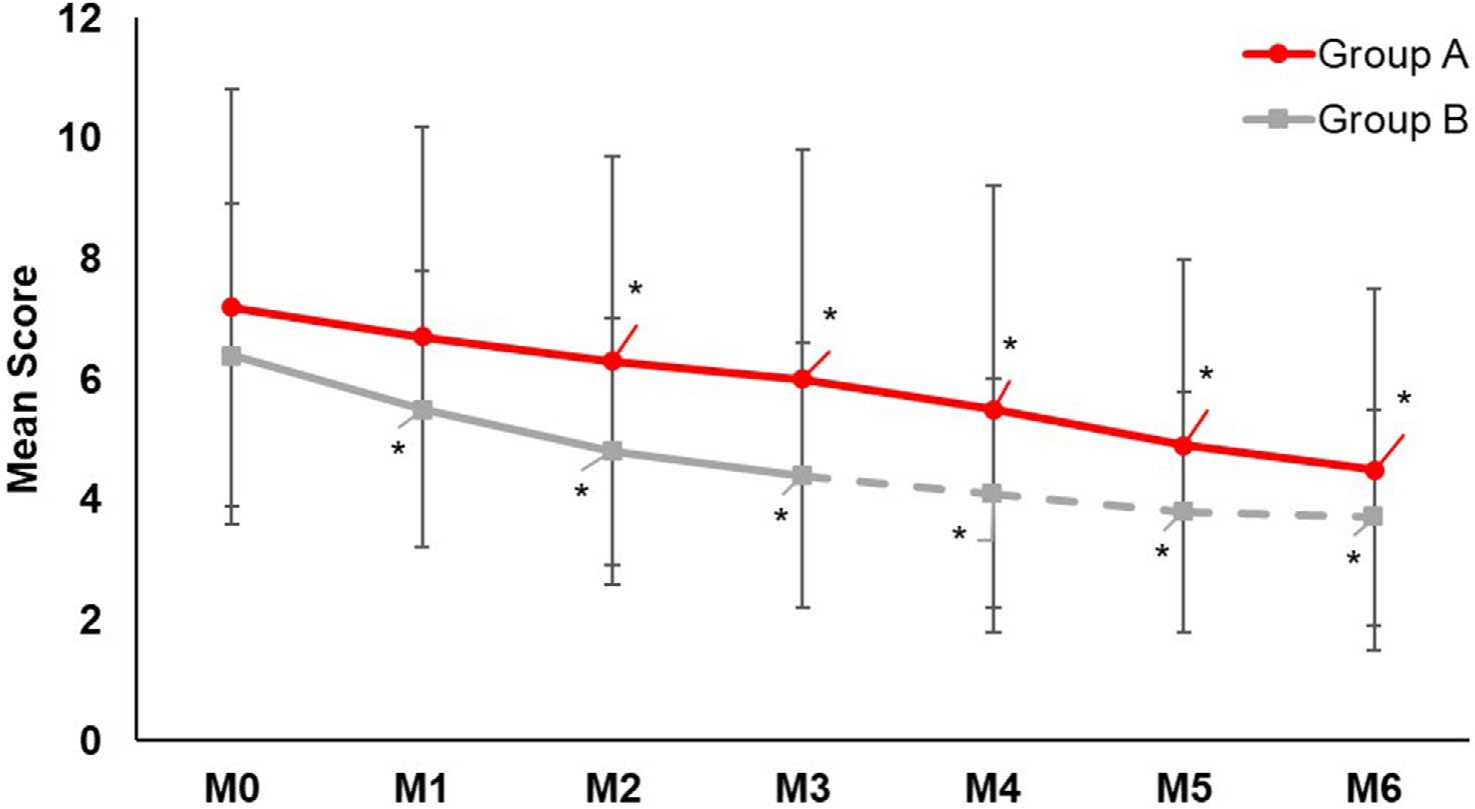
Mean mMASI score over time. The decrease of the mMASI score was statistically significant from baseline in both groups after 3 and 6 months. The between‐group difference was not statistically significant at any time point. *p ≤ 0.05.

**FIGURE 3:**
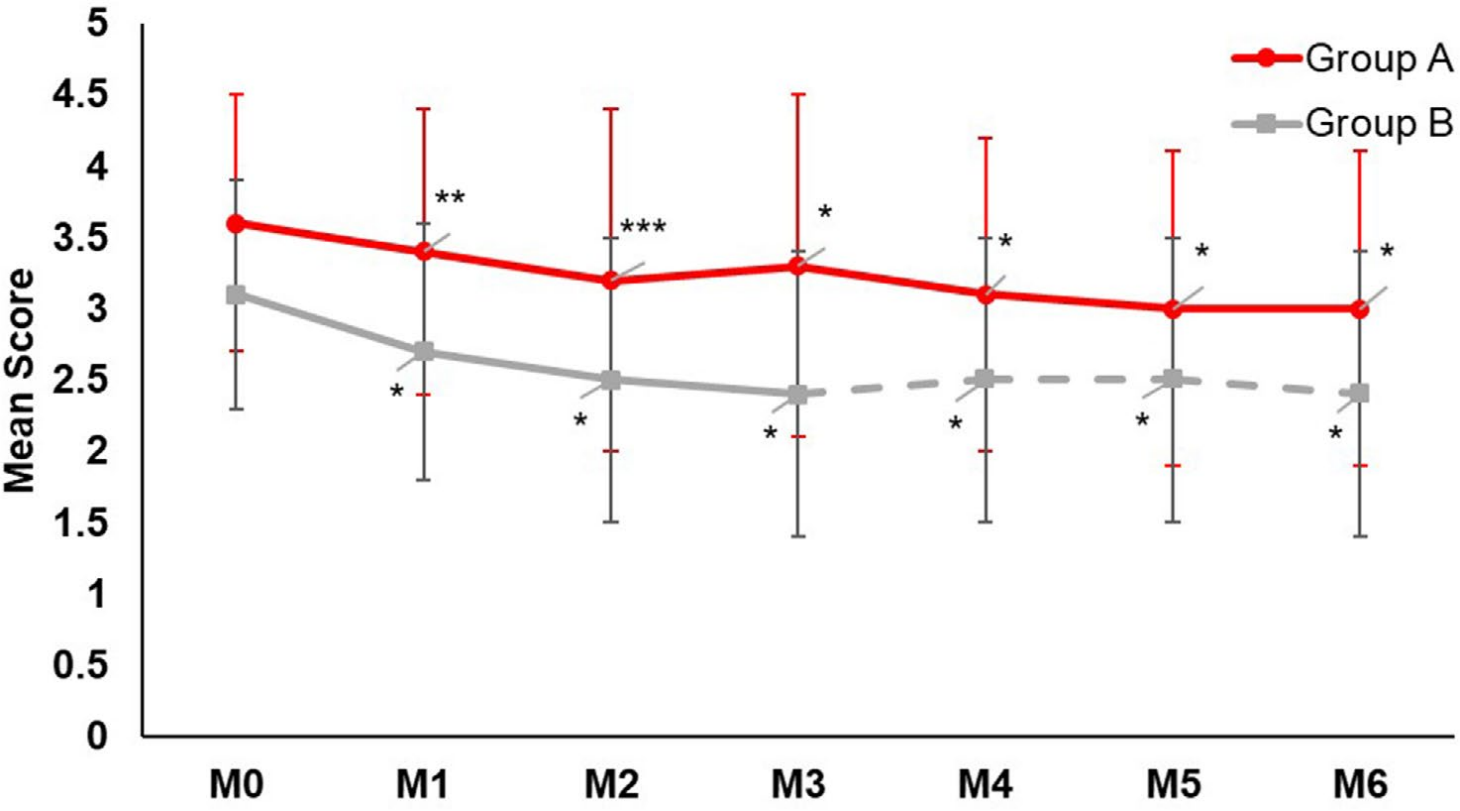
Mean hyperpigmentation IGA score over time. The decrease of the IGA score was statistically significant from baseline in both groups after 3 and 6 months. The between‐group difference was not statistically significant at any time point. *p ≤ 0.001, **p < 0.01, ***p < 0.05.

While both regimens significantly (*p* < 0.05) reduced the MASI score at Month 3 (Group A: −19.5%, Group B: −30.5%) from baseline (Figure 1), a clinically relevant but not statistically significant difference in favor of Group B was observed. Three months after the switch (month 6), both Group A (−38.1%) and Group B (−41.2%) provided a similar significant (*p* ≤ 0.05) percentage reduction of the MASI score compared to baseline, with no between‐group difference (Figure 1). Assessments for mMASI (Group A: Month 3: −17.3%, Month 6: −37.9%; Group B: Month 3: −30.9%, Month 6: −42.6%; Figure 2) and IGA of hyperpigmentation (Group A: Month 3: −8.1%, Month 6: −14.4%; Group B: Month 3: −22.9%, Month 6: −22.2%; Figure 3) provided similar significant (all *p* ≤ 0.05) results. Skin hydration significantly (*p* < 0.05) improved with the DC routine (+9.6%) from baseline, while skin hydration did not change with KT. Three months after the switch, skin hydration continued to significantly (*p* < 0.05) improve (+6.3%) with the DC routine. Group B also showed significant improvement after the switch (+6.1%).

**FIGURE 4:**
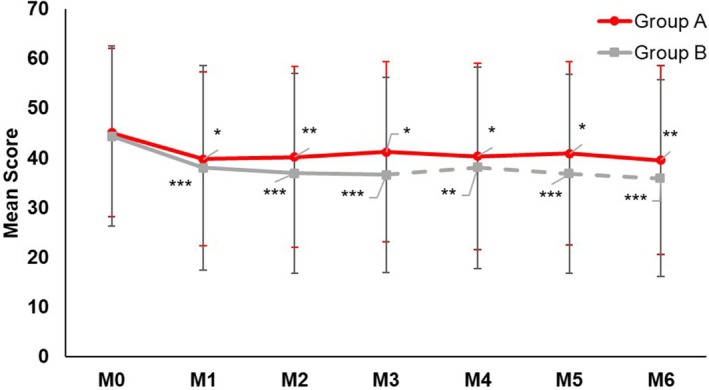
Mean MELASQOL score over time. The decrease of the IGA score was statistically significant from baseline in both groups after 3 and 6 months. The between‐group difference was not statistically significant at any time point. *p ≤ 0.05, **p ≤ 0.005, ***p < 0.001.

QoL significantly (*p* ≤ 0.05) improved in both groups after 3 and 6 months, with a decrease of the MELASQOL score in both groups (Group A: Month 3: −8.7%, Month 6: −12.4%; Group B: Month 3: −17.7%, Month 6: −19.1%; Figure 4) but with no between‐group difference. Skin radiance and fine wrinkles had also improved.

Global tolerance was similar after 3 months in both groups (Group A: 75% of subjects with no local tolerance issues vs. 72% in Group B). Three months after the switch (Month 6), 90% in Group A and 66% in Group B reported no local tolerance issues.

**FIGURE 5:**
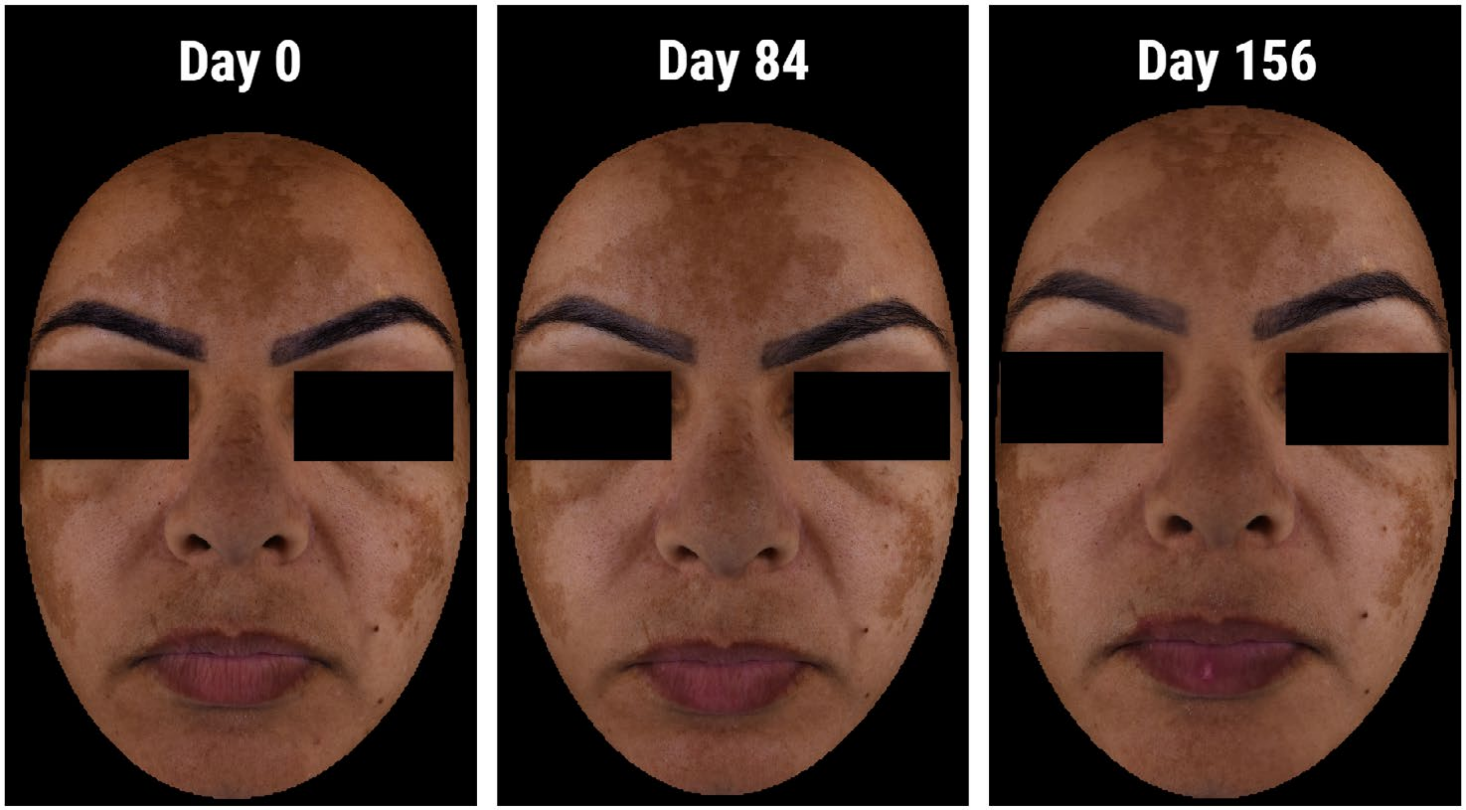
Melasma status of a subject with Phototype IV at M0, M3, and M6.

Subjects highly appreciated the cosmeticity of the DC routine (Figure 5).

## 4 Discussion and Conclusion

Despite increased research and novel approaches, the treatment of melasma remains challenging. As it is a chronic and visible skin condition impacting the subjects' quality of life, treatment requires lifelong daily and efficient care. Existing products such as KT, the gold standard, are effective but are not indicated for a prolonged period [20]. Moreover, safety, in addition to efficacy, is an important issue to consider [3]. Other commercially available (e.g., HQ in monotherapy) and “home‐made” melasma treatments are proposed. However, HQ has been red‐flagged for several safety concerns by health authorities worldwide, and KT contains HQ [21, 22, 23].

The novel tested DC routine contains vitamin B3 and 2‐mercaptonicotinyl glycine, but neither a corticosteroid nor HQ is available without the need for a medical prescription.

2‐Mercaptonicotinyl Glycine inhibits in vivo UV‐induced skin pigmentation mechanisms without impacting the subject's safety [24]. Combining this active with vitamin B3 (or niacinamide), tranexamic acid, and two other compounds enables efficient management of hyperpigmentation issues, including melasma [25, 26, 27].

When tested against KT in the herewith‐presented single‐blind randomized clinical study, the DC routine significantly (*p* < 0.05) improved melasma after 3 months of daily use. The progressive and similar decrease of MASI and mMASI scores, and the improvement of hyperpigmentation, confirm the depigmenting effect of both regimens during the first 3 months, with no significant between‐group difference, and a continued and similar improvement in both groups with no rebound once KT was replaced by the DC routine for a further 3‐month treatment period. Both the DC routine and KT improved skin radiance and fine wrinkles, thereby confirming the efficacy of retinoic acid in improving signs of skin aging.

When assessing QoL in this study, both the DC routine and KT significantly (*p* ≤ 0.05) improved subjects' QoL as early as 1 month into treatment and continued to improve QoL until Month 6.

The main limitation of this study was the single‐center design and the absence of patients of Asian ethnicity and male patients, which unfortunately prevents conclusions for these particular subgroups prone to melasma.

Despite these limitations, the DC routine can be considered as safe and as efficient as KT after 3 months of daily use, providing further and similar improvement of melasma when replacing KT for 3 additional months.

The DC routine may be proposed as an alternative to KT in melasma, either as a long‐term or as a replacement therapy after an initial 3‐month KT use.

## Author Contributions

C.D.‐B., E.C., M.F.F., E.F. and N.K. conducted the study and analyzed the data, J.R., T.C. and T.P. provided medical input. All authors validated the data, participated and validated the manuscript.

## Ethics Statement

This study received ethical approval from the Ethics Committee of the Pro‐Cardiac Hospital (6.721.734 issued on March 24, 2024).

## Conflicts of Interest

T.P., J.R. and T.C. received honoraria from Laboratoires Vichy, France; the other authors are employees of L'Oréal Group.

## Data Availability

The herewith presented data are available, upon reasonable request, from the corresponding author.
